# Exploring the miRNAs Profile in Dark-Cutting Beef

**DOI:** 10.3390/foods13060960

**Published:** 2024-03-21

**Authors:** Laura González-Blanco, Luis J. Royo, Yolanda Diñeiro, Susana García-Torres, Ana Coto-Montes, Verónica Sierra, Mamen Oliván

**Affiliations:** 1 Área de Sistemas de Producción Animal, Servicio Regional de Investigación y Desarrollo Agroalimentario (SERIDA), Ctra. AS-267, PK 19, 33300 Villaviciosa, Spain; lgblanco@serida.org (L.G.-B.); royoluis@uniovi.es (L.J.R.); yolandadg@gmail.com (Y.D.); mcolivan@serida.org (M.O.); 2 Instituto de Investigación Sanitaria del Principado de Asturias (ISPA), Av. Del Hospital Universitario, s/n, 33011 Oviedo, Spain; acoto@uniovi.es; 3 Departamento de Biología Funcional, Universidad de Oviedo, Av. Julián Clavería, 6, 33006 Oviedo, Spain; 4 Centro de Investigaciones Científicas y Tecnológicas de Extremadura (CICYTEX-La Orden), Junta de Extremadura, Guadajira, 06187 Badajoz, Spain; susana.garciat@juntaex.es; 5 Departamento de Morfología y Biología Celular, Universidad de Oviedo, Av. Julián Clavería, 6, 33006 Oviedo, Spain

**Keywords:** DFD beef, meat quality, MiRNA, oxidative stress, skeletal muscle

## Abstract

In an animal production system, different stressors may cause the depletion of muscle glycogen stores, resulting in an elevated pH at 24 h *post mortem* (pH_24_), which leads to cell metabolism alterations that affect the conversion of muscle into meat, causing meat quality defects, such as dark-cutting beef, also known as dark, firm, and dry (DFD) beef. This process may involve the alteration of small non-coding RNAs (miRNAs), which play critical regulatory roles in cellular processes. Here, we determined whether differential miRNA expression in the *Longissimus thoracis et lumborum* muscle from the Asturiana de los Valles breed at 24 h *post mortem* could serve as an early indicator of beef quality defects. Following total RNA extraction, complete miRNAome sequencing revealed 12 miRNAs that were significantly upregulated (*p* < 0.001) in DFD beef compared to the levels in CONTROL beef. These miRNAs are mainly involved in the cellular responses to redox imbalances and apoptosis. Among these, four miRNAs known to be related to oxidative stress (*bta-miR-1246*, *bta-miR-2332*, *bta-miR-23b-5p*, and *bta-miR-2411-3p*) were validated via quantitative RT-PCR. Some of their target proteins were also analyzed using Western blotting. High 70 kDa heat shock protein and low Caspase-9 expressions (*p* < 0.01) were found in DFD beef, suggesting the downregulation of apoptosis. These results suggest the importance of miRNAs in regulating stress in muscle cells during early *post mortem*, as differences in the abundance of some of these miRNAs are still observed at 24 h *post mortem*. These changes lead to an inadequate conversion of muscle into meat, resulting in meats with quality defects.

## 1. Introduction

Under different stressors affecting farming animals, muscle cell homeostasis may be disrupted, causing damage to proteins, nucleic acids (DNA and RNA), and lipids. Cells then activate mechanisms to restore cellular homeostasis or adapt to environmental conditions through growth arrest, repair, or removal of damaged macromolecules. Excessive damage may trigger programmed cell death (autophagy and apoptosis) [1,2]. These alterations in the muscle-to-meat conversion process affect final meat quality.

The depletion of glycogen stores before slaughter, which limits *post mortem* glycolysis and lactic acid formation, resulting in an abnormal muscle pH decline, is one of the first consequences of stress in animal muscle cells [3]. Muscles with an ultimate pH (pH_24_) ≥ 6 result in a beef quality defect known as dark-cutting or dark, firm, and dry (DFD) beef. DFD beef causes consumer rejection due to undesirable flavor, darker color, abnormal texture, and higher susceptibility to microbial contamination, resulting in high economic losses and food waste [4]. Despite efforts to alleviate stress during handling and transportation and enhance management practices, DFD beef remains a significant problem in several countries, with incidence rates ranging from 1% to 40% in countries such as Canada (1.3%), the US (3.2%), Brazil (4.5%), and China and Australia (>10%) [4,5]. Previous studies revealed that approximately 4% of bovine carcasses in Spain had pH_24_ > 6, which implies a considerable economic loss owing to the devaluation of the carcasses by 30–60%, resulting in significant food waste [6]. Understanding how genetics, gene expression, and regulation interact with *post mortem* biochemical processes in muscle tissue would enable a better understanding of the muscle processes that affect final meat quality and allow the identification of cellular molecules that could be used as early biomarkers of meat quality defects.

The miRNAs are small (19–24 nucleotides) non-coding RNA molecules that function as molecular rheostats that regulate gene expression in many physiological processes. In fact, miRNAs control post-transcriptional repression of target genes and can regulate the function of entire networks of genes [7]. The possibility that several miRNAs can target a single gene adds a great deal of complexity to the understanding of genetic mechanisms. Based on accumulating evidence, miRNAs can regulate gene expression in various biological processes, such as signal transduction, cell differentiation, cell proliferation, cell metabolism, and cell death [8]. There is growing evidence that miRNAs can be released from cells through two mechanisms: active secretion or passive release via membrane leakage. Once released, miRNAs can enter the bloodstream or other biological fluids in a stable cell-free form [9]. This phenomenon allows circulating miRNAs (ci-miRNAs) to facilitate communication between cells, influencing physiological and pathological pathways regardless of their distance from their point of origin [10].

Muscle-specific miRNAs, also known as myomiRNAs, regulate diverse aspects of skeletal muscle function [11]. In cattle, miRNAs have been thoroughly investigated due to their crucial role in influencing various traits related to skeletal muscle. For instance, *miR-1* and *miR-133* are muscle-specific miRNAs known to be involved in modulating muscle proliferation [12], while *miR-2373-5p* and *miR-23b-3p* are highly expressed in intramuscular fat [13]. In functional miRNA studies on skeletal muscle development in calves, *miR-148a-3p* was found to inhibit the proliferation of bovine muscle cells and promote apoptosis [14]. Moreover, *bta-miR-182*, *bta-miR-183*, and *bta-miR-338* are associated with the promotion of apoptosis and increased proteolysis; thus, they are associated with increased meat tenderness [15]. However, the relationship between different miRNAs in bovine meat and their role in the final meat quality requires further analysis.

Advances in genetics revealed the role of miRNAs in modulating cellular stress responses [16,17]. By comparing the transcriptomes of the *Longissimus thoracis* and *Semitendinosus* muscles in cows exposed to normal or limited stress conditions, Cassar-Malek et al. [18] found that variations in the degree of stress exposure led to variations in transcription factor levels. Therefore, these factors may play a role in relaying the adaptive physiological responses of cattle muscles to cope with emotional and physical stress.

Specific miRNAs expressed in response to stress in the skeletal muscle can regulate physiological networks that lead to the development of DFD beef. In a previous study, Riggs et al. [19] analyzed the differential expression of miRNAs in dark-cutting beef biopsies, obtained immediately after slaughter from castrated steers of Angus x Nellore breeds. This study identified differences in certain miRNAs that may influence the regulation of stress responses, ultimately resulting in dark-cutting beef carcasses.

Furthermore, due to their physical characteristics and small size, miRNAs are more resistant to degradation [20], making their *post mortem* analysis suitable for the identification of biological biomarkers of beef quality defects at 24 h *post mortem*, when commercial carcasses become accessible for sampling. Therefore, this study aimed to identify a differential miRNA abundance profile between muscle samples from normal pH_24_ (CONTROL) and DFD beef at 24 h *post mortem* and test their potential as reliable biomarkers of DFD beef. These biomarkers would enable the development of strategies aimed at mitigating its economic impact.

## 2. Materials and Methods

### 2.1. Muscle Sample Collection

A total of 1530 yearling bulls of the Asturiana de los Valles (AV) breed, slaughtered between 14 and 18 months of age, were monitored, complying with EU regulations, at various abattoirs in Asturias region. This native breed originates from Northern Spain and is protected by the quality label Ternera Asturiana, which holds significant importance in terms of production and economic value in Spain (MAPAMA, Madrid, Spain, 2021). After slaughter, carcasses were placed in a refrigerated chamber maintained at 3 °C until 24 h *post mortem*, when the pH (pH_24_) of the *Longissimus thoracis et lumborum* (LTL) muscle at the 13th rib level of the left half carcass was measured using a pH meter (InLab Solids Go-ISM, Mettler-Toledo S.A.E., Barcelona, Spain) calibrated with standard buffer solutions having pH values of 4.0 and 7.0 (Hamilton DuraCal™, Bonaduz AG, Switzerland).

The pH_24_ measurement was employed to classify carcasses into CONTROL (5.4 ≤ pH_24_ ≤ 5.6) or extreme DFD (pH_24_ ≥ 6.2). This threshold of 6.2 for pH_24_ ensured precise identification of defective meat [21]. Eighteen extreme DFD carcasses were identified, indicating that DFD samples accounted for 1.2% of the total monitored carcasses. For each identified DFD carcass, a carcass from the same slaughter batch and with similar origin, weight, and age was selected as a CONTROL carcass, provided its pH_24_, was normal. In total, 36 beef samples (18 extreme DFD and 18 CONTROL) were collected.

At 24 h *post mortem*, 20 g muscle samples were excised from the 13th rib level LTL muscle from the left half of 36 carcasses. These samples were snap-frozen in liquid nitrogen and then stored at −80 °C until analysis. They were used for miRNA deep sequencing, real-time qPCR, and Western blot analysis. At 48 h *post mortem*, a portion of the LTL muscle was extracted from the same left half carcasses, spanning from the 6th to the 13th rib, which were later divided into 5 steaks starting from the 6th rib in the laboratory. The first two steaks (2.5 cm each) were used for color measurement and water holding capacity (WHC) at 48 h *post mortem*. The next 3 steaks (3.5 cm each) were used for Warner–Bratzler shear force evaluation. They were vacuum-packed in polyamide 20 µm/polyethylene 70 µm bags and aged at 4 °C for 3, 7, and 14 days, respectively, and then frozen and stored at −20 °C until analysis.

### 2.2. Meat Quality Measurements

Beef color was measured at room temperature with a Minolta CM-2300d spectrophotometer (Konica Minolta Inc., Osaka, Japan) at three arbitrary spots on the exposed cut surface of each steak after 60 min of blooming. The colorimeter was set with an aperture size of 8 mm with a D65 illuminant and a 10° standard observer. The CIE lightness (L*), redness (a*) yellowness (b*) were measured and used to calculate chroma (C* = √(a*^2^ + b*^2^)) and hue angle (*h*° = arctangent (b*/a*). The average value was calculated for each sample [22].

The WHC of fresh meat was evaluated in duplicates by the drip centrifugal method developed by Jauregui et al. [23], with minor modifications [24]. Briefly, 1.5 ± 0.3 g of ground muscle was placed into a thimble created with two pre-weighted Whatman filter papers, measuring 9 and 5 cm each. The thimble was placed in a 50 mL polycarbonate tube and centrifuged at 1950× *g* for 20 min at 4 °C. After centrifugation, the thimble was extracted from the tube, the ground meat was separated from the filters, and the filters were reweighed. The centrifugal drip was reported as the percentage of weight lost from the original sample.

Warner–Bratzler shear force (WBSF) test was performed on 3.5 cm steaks aged for 3, 7, and 14-days *post mortem* as described by Díaz et al. [25]. Briefly, steaks were thawed at 4 °C for 24 h, and then cooked for 30 min at 75 °C in a water bath. After cooling, eight cores (1 cm^2^ in cross-section, with the muscle fibers parallel to the longitudinal axis of the sample) from each steak were subjected to a perpendicular cut using the TA.XT Plus instrument (Stable Micro Systems) equipped with the WB blade set HDP/WBV with a “V” slot. The load cell was 30 kg load cell and the crosshead speed of 1 mm/s. The maximum load required for total split was recorded. Results were expressed as the mean WBSF maximum load (N) value for each steak.

### 2.3. miRNA Deep Sequencing

#### 2.3.1. RNA Sequencing

Among the 36 beef samples collected, 10 samples (five pairs of DFD/CONTROL samples) were selected to identify miRNAs that differed between DFD and CONTROL beef. Samples were sent for miRNA deep sequencing to QIAGEN Genomic Services (QIAGEN GmbH, Hilden, Germany). Briefly, 25 mg of tissue samples were used for RNA extraction using miRNeasy Mini Kit (Qiagen, Barcelona, Spain (Ref:74104)), as described in the manufacturer’s instructions, with an elution volume of 40 µL. The integrity of each RNA sample was evaluated using the Agilent TapeStation in order to obtain a RINe value (RINe = RNA integrity number). All the submitted samples exhibited RINe values greater than 7, indicating the suitability of the RNA quality for NGS analysis.

QIAseq miRNA Library Kit (Qiagen, Barcelona, Spain (Ref:331502)) was employed to prepare the miRNA libraries. A total of 100 ng of RNA was converted into miRNA NGS libraries. Following adapter ligation, unique molecular identifiers (UMIs) were incorporated during reverse transcription. Complementary DNA (cDNA) was amplified via PCR (16 cycles), with PCR indices subsequently added. After PCR, the samples were purified. Quality assessment of library preparation was performed using capillary electrophoresis (Agilent DNA 1000 chip). The libraries were then grouped into equimolar ratios based on insert quality and concentration measurements. The library pools were quantified using qPCR and then sequenced using a NextSeq sequencing platform (Illumina Inc., San Diego, CA, USA), according to the manufacturer’s protocol. Using bcl2fastq2 v.2.20.0.422 software (Illumina Inc., San Diego, CA, USA), the raw data were demultiplexing, resulting in the generation of FASTQ files for individual samples. The expression level of each miRNA was assessed according to read frequency, and the results were normalized to the number of reads per million (RPM) using the following formula: RPM = (miRNA read number/total mapped miRNA reads per library) × 10^6^.

All primary analyses were conducted using CLC Genomics Server 21.0.4. The workflow QIAseq miRNA Quantification of CLC Genomics Server is used to map the reads to miRBase vs.22. In summary, the reads are processed by (1) trimming of the common sequence, UMI, and adapters, and (2) filtering of reads with length < 15 nt or length > 55 nt. Subsequently, deduplication is performed using their respective UMI. Reads are organized into UMI groups when they (1) start at the same position based on the end of the read to which the UMI is ligated (i.e., Read2 for paired data), (2) are from the same strand, and (3) possess identical UMIs. Groups that contain only one read (singletons) are merged into non-singleton groups if the singleton’s UMI can be transformed into a UMI of a non-singleton group by introducing an SNP (the biggest group is chosen). Reads that do not map to miRBase, either with perfect matches or as isomiRs (maximum two mismatches and/or alternative start/end positions of 2 nt), are mapped to the *Bos taurus* genome ARS-UCD1.2 with annotation ENSEMBL ARS-UCD1.2 vs.104. This process is conducted using the RNA-Seq Analysis workflow of CLC Genomics Server with standard parameters.

#### 2.3.2. Identification of Candidate Reference miRNAs for qPCR Normalization

MiRNAs used as candidates for normalization in qPCR were pre-selected using the NormFinder algorithm. NormFinder, an ANOVA-based algorithm, was employed to assess the overall variation in candidate reference genes across subgroups, considering both intergroup and intragroup variations [26]. This algorithm assigns a stability value to each candidate gene, directly indicating the estimated variation in expression. Consequently, it enables users to assess systematic errors introduced during normalization with the gene.

#### 2.3.3. Identification of miRNAs with Different Levels between the CONTROL and DFD Groups

The “Empirical analysis of Differential Gene Expression (DGE)” algorithm from CLC Genomics Workbench 21.0.4 was employed for differential expression analysis in the ten samples sequenced (five per group). This algorithm applies the “Exact Test” for comparisons between two groups, following the approach described by Robinson and Smyth [27] and incorporated into the EdgeR Bioconductor package [28]. In all unsupervised analyses, only miRNAs with a minimum count of 10 across all samples were included. A variance stabilizing transformation was applied to the raw count matrix using the *vst* function from the R package DESeq2 version 1.28.1.

### 2.4. Validation of Candidate miRNAs via Quantitative Real-Time PCR Analysis (RT-qPCR)

#### 2.4.1. RT-qPCR

A subset of miRNAs with significant differences between CONTROL and DFD meat was validated using RT-qPCR in the whole set of samples (*n* = 36). Total RNA was extracted from the LTL muscles of the CONTROL (*n* = 18) and the DFD (*n* = 18) samples using mirVana miRNA Isolation Kit (Applied Biosystems, Foster City, CA, USA), according to the manufacturer’s protocol. The synthesis of cDNA was performed using total RNA and the TaqMan Advanced miRNA cDNA Synthesis Kit (Thermo Fisher Scientific, Whaltham, MA, USA) and stored at −20 °C for further analysis. The miRNA levels were determined via RT-qPCR using a StepOne thermocycler (Applied Biosystems, Foster City, CA, USA). The final reaction solution contained 4 μL of RNase-free water, 10 μL of 2× TaqMan Fast Advanced Master mix (Thermo Fisher Scientific Whaltham, MA, USA), 1 μL of 20× TaqMan Advanced miRNA Assay (Thermo Fisher Scientific Whaltham, MA, USA), and 5 μL of cDNA (dilution 1:10). The following thermocycling protocol was applied: 95 °C for 20 s, followed by 40 cycles at 95 °C for 1 s and 60 °C for 20 s. PCR was performed in duplicate, allowing for a maximum discrepancy of 0.5 threshold cycles between duplicates.

#### 2.4.2. GeNorm Analysis: Selection of Stable Reference miRNA

GeNorm, a popular algorithm for identifying stable reference genes from a set of tested candidate reference genes under specified experimental conditions [29], was applied to calculate the optimal number and selection of reference genes for normalization using the miRNAs outlined in Section 2.3.2 in 36 beef samples of different meat quality grades (CONTROL vs. DFD).

#### 2.4.3. Normalization of miRNA Levels

MiRNA levels were standardized using the geometric average of the reference miRNAs selected by geNorm, estimated by the QBase+ software (v 3.1, Biogazelle, Zwijnaarde Belgium) [30], and presented as base log10. The mean miRNA levels were compared between CONTROL and DFD beef using Student’s *t*-test in SPSS software (v.22.0; SPSS Inc., Chicago, IL, USA). The results are expressed as mean ± standard error of the mean (SEM). Statistical significance was set at *p* < 0.05.

### 2.5. Extraction of Sarcoplasmic Proteins

The sarcoplasmic proteins extraction was performed using 0.5 g of muscle per sample, which were homogenized in 4 mL of TES buffer (10 mM Tris pH 7.6, 1 mM EDTA pH 8.0, 0.25 M sucrose, and 0.6% protease inhibitor cocktail (P8340, Sigma-Aldrich Co., St. Louis, MO, USA) with a Polytron PT1200 E (Kinematica Inc., Luzern, Switzerland) twice for 15 s at maximum speed. The homogenate was centrifuged for 20 min at 20,000× *g* at 4 °C. To ensure consistency and minimize variability, the supernatants of each individual DFD and CONTROL sample were randomly split into two groups and combined, with each group containing half the pooled samples. Thus, two sets of CONTROL and two sets of DFD samples were obtained, following the procedure outlined by González-Blanco et al. [24]. Three replicates were prepared for each extracted sample and pooled as described above. Samples were stored at −80 °C for further analysis. Bradford method was used for estimating the protein content.

### 2.6. Western Blotting

The homogenized tissue (90 µg protein) was mixed with Laemmli sample buffer (Bio-Rad Laboratories, Inc., Hercules, CA, USA) and denatured by boiling at 100 °C for 5 min. The samples were fractionated via SDS-PAGE at 200 V, followed by the transfer of proteins onto polyvinylidene fluoride membranes (Immobilon TM-P; Millipore Corp., MA, USA) at 350 mA. The membranes were blocked overnight at 4 °C with a solution of 10% (*w*/*v*) of skim milk dissolved in Tris-buffered saline (TBS) (50 mM Tris-HCl and 150 mM NaCl, pH 7.5), followed by incubation overnight at 4 °C with the respective primary antibodies: Caspase-9 (CASP9) (ab202068, Abcam, Cambridge, UK) and heat shock protein 70 kDa (HSP70) (ab2787, Abcam), which were diluted in TBS buffer supplemented with 5% (*w*/*v*) albumin (BSA). Once blocked, membranes were washed with TBS-T (50 mM Tris-HCl, pH 7.5, 150 mM NaCl, and 0.05% Tween-20) three times. Then, membranes were incubated with the appropriate horseradish peroxidase-conjugated secondary antibody (Cell Signaling, Danvers, MA, USA) for 1 h at 25 °C. Horseradish peroxidase was used as a substrate for the detection of immunoconjugates (WBKLS0500; Millipore Corp., Darmstadt, Germany). GeneTools software v 4.3.10.0 (Syngene, Cambridge, UK) was used to quantify the optical densities of the bands. The densitometry results are expressed as semi-quantitative optical density (in arbitrary units) of blot bands, and owing to variations in the typical constitutive protein levels (GAPDH, β-actin, and α-tubulin), the results were normalized to Ponceau, which served as the loading control [31]. Three replicates were employed for each sample pool.

### 2.7. Statistical Analysis of Quality Attributes and Western Blot Data

SPSS software (vs. 22) was used for statistical analysis. The normality of the data was verified using the Kolmogorov–Smirnov test. The effect of pH_24_ sample type on meat quality traits (drip loss and color attributes (L*, a*, b*, C* and *h°*) and CASP9 and HSP70 expression levels were analyzed in 18 samples from each type (CONTROL and DFD) by an independent samples *t-test*. The *t-test* value was calculated as:

$$T\text{-value}=\frac{{\bar{X}}_{1}-{\bar{X}}_{2}}{\sqrt{\frac{{S_{1}}^{2}}{n_{1}}+\frac{{S_{2}}^{2}}{n_{2}}}}$$

where ${\bar{X}}_{1}$ and ${\bar{X}}_{2}$ are the means of the two independent groups (CONTROL and DFD); *S*_1_ and *S*_2_ are the sample variances of each of the two groups; *n*_1_ and *n*_2_ are the sample sizes of the two groups.

The *t-statistic* follows a Student’s t-distribution under the null hypothesis, with statistical significance set as *p* < 0.05.

WBSF, measured at different *post mortem* times, was analyzed by the general linear model, which included pH_24_ sample type (T), *post mortem* time (t), and their interaction as fixed factors:

Y_ij_ = μ + T_i_ + t_j_ + (T_i_ × t_j_) + Ɛ_ij_

where Y_ij_ = dependent variable (WBSF), μ = population mean, T_i_ = pH_24_ sample type (_i_ = 1,2), t_j_ = *post mortem* time (_j_ = 1,2,3) and Ɛ_ij_ = residual random term.

Significant differences among *post mortem* times were assessed using Tukey’s test (Games–Howell test when variances were not homogeneous) with statistical significance set at *p* < 0.05.

## 3. Results

### 3.1. Meat Quality Traits

Table 1 shows the results of meat quality traits from the 18 CONTROL and 18 DFD beef samples analyzed. The DFD meat displayed significantly lower drip loss (*p* < 0.001) and decreased values of L* (*p* < 0.01), a* (*p* < 0.001), b* (*p* < 0.01), and C* (*p* < 0.001), suggesting a darker, brownish, and more saturated color.

**Figure 1:**
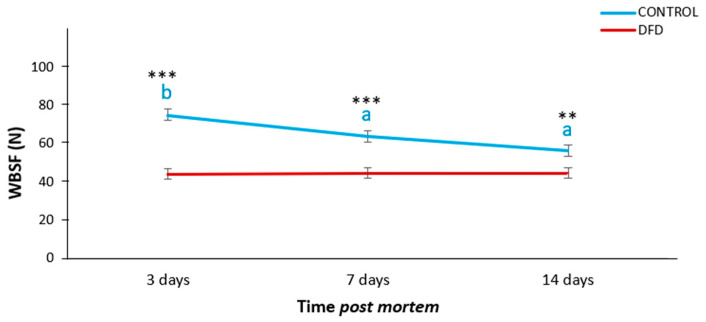
*Post mortem* evolution of Warner-Braztler shear force (WBSF) showing the meat tenderization pattern of CONTROL (blue line) and DFD (red line) samples. Different blue lowercase letters indicate significant differences in WBSF along *post mortem* for control samples (*p* < 0.05). Asterisks indicate significant differences between CONTROL and DFD at the same storage time. *** *p* < 0.001; ** *p* < 0.01.

DFD samples exhibited significantly lower values of WBSF than CONTROL samples at 3 (*p* < 0.001), 7 (*p* < 0.001), and 14 (*p* < 0.01) days *post mortem*. Moreover, DFD showed constant values of meat toughness along meat maturation while CONTROL samples exhibited a normal *post mortem* tenderization trend, with a significant decrease (*p* < 0.001) in meat toughness during aging (Figure 1).

### 3.2. miRNA Levels in CONTROL and DFD Beef

Sequencing of the miRNAs from RNA extracted from the LTL of CONTROL (n = 5) and extreme DFD (n = 5) beef samples at 24 h *post mortem* was performed. miRNA reads were analyzed using miRDeep2 software v 2.0.1.3 and mapped to the *Bos taurus* (bta) genome. No significant differences were found between the total reads of the CONTROL and DFD beef (*p* = 0.230, Table 2). Almost half of the reads were from small RNAs, the most abundant of which were protein-coding RNA, in both beef groups. CONTROL and DFD beef showed significant differences in the percentages of all small RNAs, except for miscellaneous RNAs (misc-RNAs), small nuclear RNAs (snRNAs), and bacterial small RNAs (sRNAs) (Table 3). Ribosomal RNAs (rRNAs), small nucleolar RNAs (snoRNAs), miRNAs, and small Cajal body-specific RNAs (scaRNAs) were more abundant in CONTROL beef than those in DFD beef, whereas protein-coding RNAs and large non-coding RNAs (lncRNAs) showed the opposite trend.

The miRNA levels were significantly different (*p* < 0.01) between the CONTROL and DFD beef (Table 3) exhibiting DFD lower abundance levels.

### 3.3. Identification of Differentially Expressed miRNAs via Sequencing

Owing to sequencing, 1028 miRNAs were identified in the LTL muscle. Among them, only 12 were significantly different with a Log_2_ fold change >1, FDR *p*-value < 0.01 and Bonferroni corrected *p*-value < 0.05 between CONTROL and DFD (Table 4).

**Figure 2:**
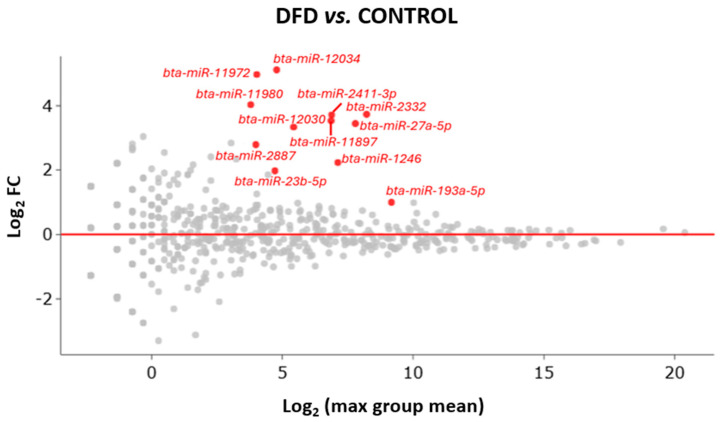
MA plot of miRNA differential expression, showing the Log_2_ fold change (y-axis) and the average expression (x-axis) across all samples. Significant changes in genes (>1 or <−1 Log_2_ fold change) from DESeq2 (*p* < 0.05) are highlighted in color. Upregulated miRNAs are represented in red dots, while miRNAs with no significant changes in expression are represented in grey dots.

All differentially expressed miRNAs were upregulated in the DFD group compared with those in the CONTROL group (Figure 2).

### 3.4. Validation of Differentially Expressed miRNAs between CONTROL and DFD Meat

#### 3.4.1. Selection of Reference miRNAs

Eight reference candidate miRNAs with high stability values obtained using the NormFinder algorithm were selected and tested via RT-qPCR in CONTROL (*n* = 18) and DFD (*n* = 18) samples. No significant differences in expression levels were found between the two groups (Table S1). Therefore, these eight putative reference miRNAs were selected and reassessed as normalizers using geNorm.

**Figure 3:**
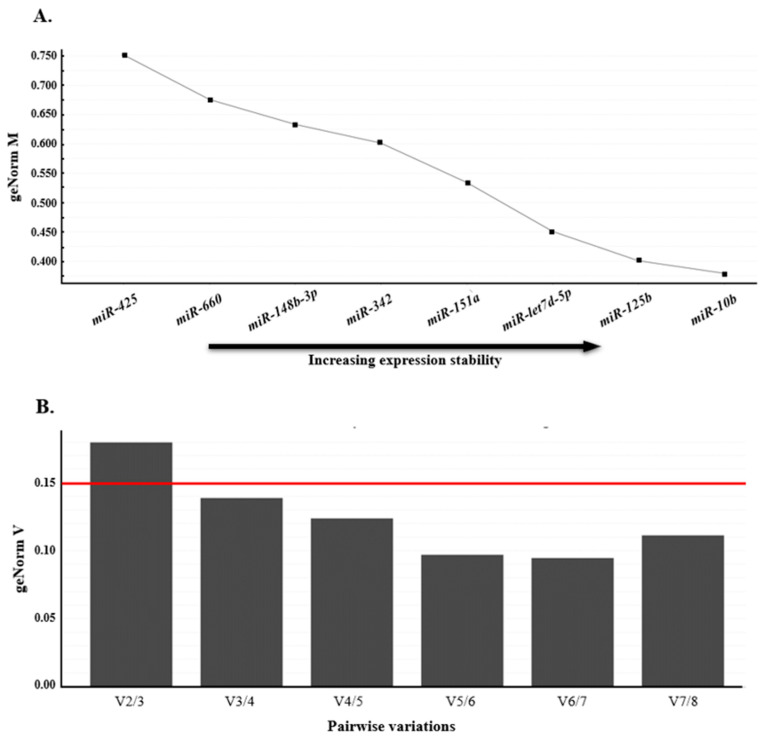
GeNorm analysis of qPCR-based candidate reference genes. (**A**) Genes with lower geNorm M values were considered more stable and the gene with an M value <0.5 was accepted as appropriate reference genes. Thus, *miR-let7d-5p*, *miR-125b*, and *miR-10b* were acceptable reference genes in this study. (**B**) The optimal number of reference genes required for normalization was determined based on pairwise variation (geNorm V value of n/n + 1) and a value <0.15 indicates the minimum number (n) of genes. Red line indicates the V value threshold. In this study, a combination of three reference genes was sufficient for normalization, as V3/4 was 0.139.

Using the average pairwise variation among all tested genes, geNorm computes a stability value M, followed by iteratively excluding the least stable gene until only the two most stable genes remain (as illustrated in Figure 3A). Genes with the highest M values exhibited the least stable expression, whereas those with the lowest M values exhibited the most stable expression. In this study, *miR-let7d-5p*, *miR-125b*, and *miR-10b* had the lowest M values, indicating that these miRNAs had the most stable expression levels. In addition, geNorm was used to determine a normalization factor (VNF value), which is used to determine the optimal number of reference genes. A VNF value of 0.15 was set as the threshold for appropriate normalization. Once this threshold was reached, additional reference genes were not required [29]. Our analyses showed that V3/4 was <0.15, indicating that the combination of these three miRNAs was the most suitable reference normalization factor (Figure 3B).

Using a combination of multiple normalizers may enhance the accuracy of qPCR quantification compared to using a single reference gene. Hence, we propose the use of a panel of miRNAs, including *miR-let7d-5p*, *miR-125b*, and *miR-10b*, as reference genes for evaluating DFD meat.

#### 3.4.2. miRNAs Selected for RT-qPCR Validation

After conducting a literature review to identify the differentially expressed miRNAs discovered through sequencing and to ascertain their biological function and analyzing their suggested target genes as indicated by TargetScan v 8.0, four miRNAs (*bta-miR-1246*, *bta-miR-2332*, *bta-miR-23b-5p*, *and bta-miR-2411-3p*) were selected for RT-qPCR validation. These miRNAs were found to be associated with oxidative stress, heat shock proteins, and the repression of apoptotic processes, all of which are known to influence the muscle-to-meat conversion process and, consequently, the development of DFD. Therefore, RT-qPCR was conducted to validate the expression levels of these miRNAs in all 36 samples collected in this study (18 DFD and 18 CONTROL samples).

**Figure 4:**
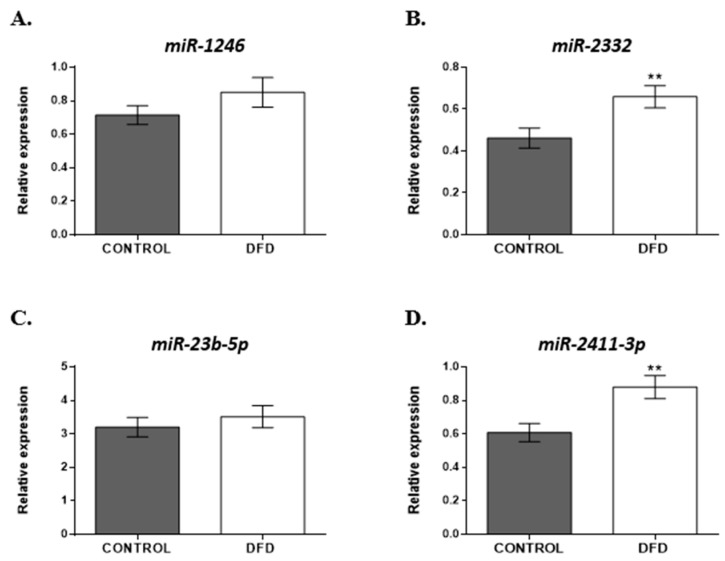
Expression levels of (**A**) *bta-miR-1246*, (**B**) *bta-miR-2332*, (**C**) *bta-miR-23b-5p*, and (**D**) *bta-miR-2411-3p* in CONTROL (grey bar) and DFD (white bar) beef samples were measured using RT-qPCR. The quantitative results are presented as mean ± SEM. **, *p* < 0.01.

Based on the analysis of the normalized miRNA levels, two miRNAs, *bta-miR-2332* and *bta-miR-2411-3p*, showed significantly greater expression (*p* < 0.01) in the DFD samples (Figure 4B,D) compared to CONTROL. Although *bta-miR-1246* and *bta-miR-23b-5p* tended to have high levels in the DFD group, the difference was not statistically significant between the two groups (Figure 4A,C).

### 3.5. Putative Target Genes and Functional Analysis

To determine the implications of the differentially expressed miRNAs on the final beef quality in the DFD and CONTROL groups, the potential target genes of *miR-2332* and *miR-2411-3p* were identified using TargetScan vs 8.0 (Table 5). Among the 3055 potential target genes proposed by TargetScan for *miR-2332*, some of them such as HSPA12B, HSPBAP1, HSPA4, HSBP1, HSPH1, DNAJA1, DNAJB9, and DNAJC10 are involved in the expression of heat shock proteins (HSPs) that play an important role in protecting cells and organisms from oxidative damage and apoptosis and therefore may impact muscle-to-meat conversion. Moreover, for *miR-2411-3p*, TargetScan identified 3382 target genes, some of which were genes that play important roles in the regulation of oxidative stress (GSR, GPX5, SERP1, and HSPA2) and apoptosis (CLU, TP53AIP1, CASP2, and CASP9), as shown in Table 5.

### 3.6. Protein Expression in Control and DFD Beef Based on the Validated miRNA Targets

Western blot analyses were performed to determine the expression levels of HSP70 and CASP9 proteins related to oxidative stress and cell death (apoptosis), which are some of the target genes regulated by *miR-2332* and *miR-2411-3p* gene targets.

**Figure 5:**
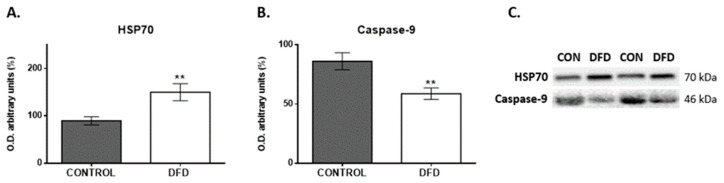
Western blot analysis of proteins related to stress response and apoptosis markers in CONTROL (grey bar) and DFD (white bar) beef samples. Bar chart depicting the semiquantitative optical density (O.D.) (arbitrary units) of the blot bands of (**A**) 70 kDa heat shock protein of (HSP70) and (**B**) Caspase-9. (**C**) Representative immunoblots. Data are presented as mean ± SEM. **, *p* < 0.01.

The results revealed greater levels of HSP70 (*p* < 0.01; Figure 5A) and significantly lower levels of Caspase-9 (*p* < 0.01; Figure 5B) in DFD beef than those in CONTROL beef.

## 4. Discussion

Animal genetics, nutrition, and handling, along with the carcass *post mortem* processing, play critical roles in determining the final meat quality. Several genes have been reported to be associated with intramuscular fat, meat tenderness, and *post mortem* proteolysis [32]. Understanding the role of regulatory elements that can promote or inhibit gene expression has become increasingly important for understanding how cells and tissues respond to changes related to metabolism and environment. miRNAs are regulatory molecules that affect post-transcriptional gene expression. miRNAs normally act as negative regulators of gene expression; however, substantial evidence suggests that miRNAs can also activate gene expression [33].

Different studies have identified miRNAs in ovine [34] and bovine [34,35] skeletal muscles, which are involved in the regulation of many different metabolic processes. Moreover, several miRNAs have been identified in the skeletal muscles of various livestock species that act as key regulators of quality trait acquisition and can therefore be considered as meat quality biomarkers [15,36]. From the moment the animal is slaughtered until the death of all its muscle cells, a period elapses during which the cells retain their ability to trigger different mechanisms to restore homeostasis. Therefore, it is reasonable to suggest that miRNAs may regulate the metabolic processes that control the *post mortem* muscle-to-meat conversion and that differences in their expression levels could allow for distinguishing meats of normal quality and DFD during early *post mortem* stages. This means that the miRNA pattern could serve as an early biomarker of DFD meats, which could aid in the development of strategies to prevent or reduce these defects, thus mitigating their economic impact.

In the current study, 1530 yearling bulls of AV were monitored, revealing 18 animals with pH_24_ ≥ 6.2, resulting in DFD quality defects. When comparing these carcasses with normal pH_24_ (CONTROL) carcasses, significant differences (*p* < 0.05) in beef quality attributes such as color, drip loss, and WBSF were observed. Meat with high pH_24_ was darker and showed lower drip loss and lower WBSF than CONTROL, agreeing with previous studies of this condition in different breeds and from diverse production systems [37,38].

A total of 1028 miRNAs were identified in the LTL muscle. Among these, 12 miRNAs were found to be differentially expressed between the CONTROL and DFD groups, with a high FDR probability (*p* < 0.05) in the sequencing analysis (Table 3) after normalization. To the best of our knowledge, there is only one previous study dealing with the differential expression of miRNAs in dark-cutting beef [19]. They found 29 differentially expressed miRNAs in muscle biopsies from *Longissimus Lumborum* collected immediately after the animal’s slaughter, but after multiple testing corrections only a single miRNA, *bta-miR-2422*, was identified at an FDR probability of *p* < 0.054. None of the miRNAs found in both studies coincide, which could be due to differences in the miRNA expression at different *post mortem* times used for the analysis (0 to 24 h *post mortem*). In fact, forensic studies in humans and rats have found that the expression of certain miRNAs decays as the *post mortem* interval increases, while there are other miRNAs whose expression increases *post mortem*, suggesting an upregulation of miRNAs needed for the body decomposition process [39].

Most of the differentially expressed miRNAs identified in this study were related to alterations in cellular metabolism and cellular responses to stress and apoptosis. Apoptosis, or programmed cell death, is a complex process that requires the activation of cysteine proteases called caspases, which cleave cellular proteins that are critical for dismantling dying cells. The relationship between apoptosis and meat quality was first proposed by Ouali et al. [40] and has since been the subject of extensive investigation. Previous studies have shown that some miRNAs, such as *bta-miR-182*, *bta-miR-183*, and *bta-miR-338*, may promote apoptosis and increase proteolysis, which leads to increased meat tenderness [15]. Moreover, recent studies have highlighted the crucial role of these processes in muscle tissues in response to oxidative stress after animal exsanguination and their influence on the muscle-to-meat conversion process [1]. Therefore, based on their biological function, four of the differentially abundant miRNAs found in the present study, *bta-miR-2332*, *bta-miR-2411-3p*, *bta-miR-1246*, and *bta-miR-23b-5p*, were chosen for further validation via RT-qPCR on the entire sample set (n = 36 samples) collected (18 DFD and 18 CONTROL). They were chosen due to their association with oxidative stress and the repression of apoptotic processes and thus could play an important role in the development of DFD defects. The results showed a greater abundance of *miR-2332* and *miR-2411-3p* (*p* < 0.01) in DFD beef samples at 24 h *post mortem.*

The upregulated expression of *miR-2332* has been observed during heat stress in cattle as this miRNA specifically targets heat shock-responsive genes, especially members of the HSP family [41]. TargetScan was used to identify several HSPs, such as heat shock 70 kDa protein 12B (HSPA12B), heat shock 27 kDa associated protein 1 (HSPBAP1), heat shock 70 kDa protein 4 (HSPA4), heat shock factor binding protein 1 (HSBP1), heat shock 105/110 kDa protein 1 (HSPH1) and some DnaJ (Hsp40) homolog, subfamily A (DNAJA1), B (DNAJB9), and C (DNAJC10), as potential target genes of this miRNA (Table 5).

The family of 70 kDa heat shock proteins (HSP70) is activated in reaction to oxidative stress. The increase in HSP70 levels inhibits caspase activity, thereby protecting cells from apoptosis. The synthesis of HSP70 in stressful situations serves a protective function in maintaining metabolic homeostasis and the structural integrity of muscle cells against damage, consequently decelerating the muscle aging rate and reducing the breakdown of myofibrillar proteins [40]. Previous proteomic research has documented differential HSP70 expression in muscles with variable meat quality traits [42]. Accordingly, in the present study, greater expression levels of HSP70 in Western blot and greater abundance of *miR-2332* in RT-qPCR were found in DFD samples at 24 h *post mortem*, which aligns with the results of Yadav et al. [43], who found a significant positive correlation (*p* < 0.01) between *miR-2332* expression and HSP70 (0.998) during heat stress in buffalo heifers. This result indicates that *miR-2332* may activate the expression of its target genes. However, further studies are necessary to understand the inhibitory and modulatory effects of miRNAs on key genes involved in the stress response in cattle.

Regarding *miR-2411-3p*, previous studies in yak and cattle yaks revealed that this miRNA regulates two important genes, clusterin (CLU) and hydroxy acyl glutathione hydrolase (HAGH). CLU is a multifunctional protein that plays important roles in lipid transport, cell adhesion, programmed cell death, and the complement cascade. Many cell types respond to cytotoxic stress signals by upregulating the CLU gene, which has been widely accepted as a marker of cell death [44]. However, alternative splicing of this gene results in the formation of three different proteins: a nuclear isoform (nCLU), which promotes cell death, and two secreted isoforms (sCLU), which prevent cell death [45]. Therefore, further studies on the function of *miR-2411-3p* in relation to its impact on cell death and final meat quality are required. HAGH is the second important target gene of *miR-2411-3p* and plays a significant role in glutathione (GSH) accumulation and regulation [46]. The greater abundance of *miR-2411-3p* in DFD may be related to HAGH downregulation and therefore, to lower GSH levels in DFD muscle cells. Consistently, previous results from our group revealed lower catalase activity and greater oxidative damage to lipids in DFD beef [47]. Similarly, Chen et al. [48] and Delles et al. [49] reported a reduction in antioxidant defense enzyme activities in stressed pigs and broilers, respectively, compared to non-stressed pigs. Apart from these, other important target genes of *miR-2411-3p* identified using TargetScan (Table 5) were glutathione reductase (GSR), glutathione peroxidase 5 (GPX5), stress-associated endoplasmic reticulum protein 1 (SERP1), heat shock 70 kDa protein 2 (HSPA2) related to oxidative stress homeostasis, tumor protein p53-regulated apoptosis-inducing protein 1 (TP53AIP1), caspase 2 (CASP2), and caspase 9 (CASP9) related to apoptosis.

Caspase-9 is a well-known initiator caspase that triggers the mitochondrial apoptotic pathway in response to cellular stress during early *post mortem*, and subsequently activates caspase-3 to execute a cell death program [50]. Previous studies have suggested a delay or decrease in apoptosis in DFD beef [51], which may lead to abnormal tenderization and quality defects in meat. CASP9 expression was analyzed via Western blotting, revealing low expression (*p* < 0.01) in DFD beef at 24 h *post mortem*, supporting the downregulation of apoptosis in this group. These results suggest a relationship between a greater abundance of *miR-2411-3p* and the downregulation of CASP9.

The other two differentially abundant miRNAs that were validated were *bta-miR-1246*, and *bta-miR-23b-5p.* Although they showed a tendency to be more abundant in the DFD group, none of them exhibited a statistically significant difference during validation. *miR-1246* is implicated in the regulation of cell survival, inflammation, and apoptosis. Moreover, this miRNA is documented to exhibit elevated expression levels in the blood plasma and serum of cattle subjected to heat stress. It exerts its protective effect against cell death by suppressing the expression of its target genes, PCBP2 and CREBL2, which play crucial roles in apoptosis signaling pathways [52].

On the other hand, *miR-23b-5p* participates in the regulation of cell proliferation, inflammation, and apoptosis [53]. Liu et al. [54] confirmed that the overexpression of *miR-23b* resulted in a striking downregulation of the proapoptotic gene, POX, to evade apoptosis. You et al. [55] found that *miR-23b-5p* was upregulated in myocardial tissues under hypoxia, leading to decreased mitochondrial content and impaired oxidative respiration. Similarly, sudden cessation of blood flow after cattle exsanguination causes hypoxia in skeletal muscle cells; therefore, *miR-23b-5p* could exert a similar effect.

In addition to the four miRNAs validated using RT-qPCR, *miR-193a-5p*, which was identified via sequencing (Table 4) but not validated in this study, was also related to significant suppression of oxidative stress-induced apoptosis, as revealed by increased anti-apoptotic protein (Bcl-2) and decreased proapoptotic Bax expression in prostate cancer cells [56].

The remaining differentially expressed miRNAs found in the deep sequencing have been involved in different biological processes. *miR-27a-5p* acts as a negative regulator of adipocyte differentiation and lipid metabolism and a positive regulator of cell proliferation [57]. *miR-2887* is involved in the regulation of immune and inflammatory responses [58]. Also, *miR-11987* and *miR-11972* have been shown to be involved in the autophagic process by negatively regulating their target genes, RAC3 and CLN7 [58,59]. RAC3 is a negative regulator of autophagy; therefore, its downregulation by *miR-11972* could promote autophagy in DFD beef. Consistently, Brandenstein et al. [60] observed that the absence of the lysosomal membrane protein, CLN7, in CLN7-knock-out mice, led to lysosomal dysfunction and impaired autophagy, as suggested by altered autophagic flux with decreased Beclin-1, increased autophagosome marker LC3-II, and the presence of p62 and ubiquitin-positive protein aggregates. Previous studies by our group revealed a significant increase in some autophagic biomarkers (Beclin-1 and LC3-II) in DFD beef [51] at 24 h *post mortem*, which could be at least partially influenced by *miR-11987* and *miR-11972.*

Finally, in the case of *miR-11980*, *miR-12034,* and *miR-12030* no information of their function was found in the scientific literature reviewed.

**Figure 6:**
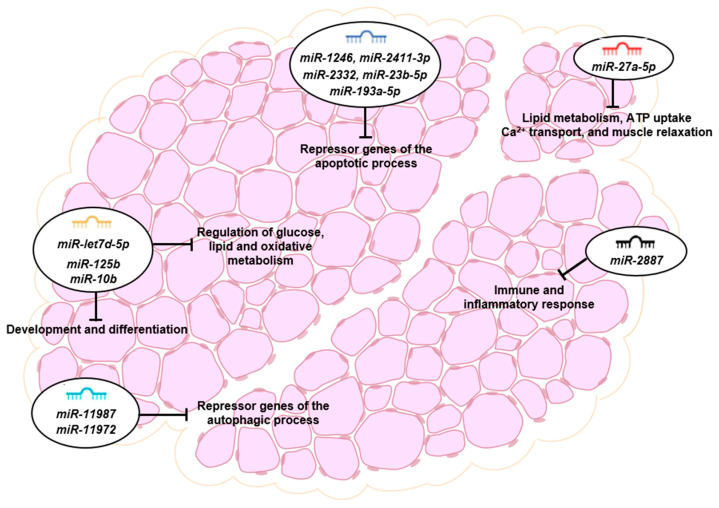
microRNA functions and target genes in the *post mortem* skeletal muscle.

The key miRNAs differentially expressed at 24 h *post mortem* identified in our study may provide putative regulatory candidates for future research on beef quality traits and provide insights into the main biological pathways and regulatory molecules involved in the muscle-to-meat conversion process (Figure 6).

## 5. Conclusions

The present study provides relevant information on the effects of genetic regulatory mechanisms on the final meat quality. We found that *bta-miR-2332* and *bta-miR-2411-3p* are regulatory molecules that are differentially abundant at 24 h *post mortem* in CONTROL and DFD beef, thereby serving as potential biomarkers of alterations in physiological responses that result in meat quality defects. Early identification of these problems could reduce the impact of DFD meat, which continues to cause significant economic losses and food waste. To be able to use these miRNAs as DFD meat biomarkers, it would be interesting to know their levels at earlier *post mortem* times and their evolution during meat aging.

## Figures and Tables

**Table 1:** The effect of pH_24_ sample type (CONTROL and DFD) on drip loss and color parameters.

Variable	Time *post mortem*	CONTROL (5.4 ≤ pH_24_ ≤ 5.6)	DFD (pH_24_ ≥ 6.2)	SEM	*p*-Value
Drip loss (%)	48 h	30.98	21.84	0.79	0.000
Meat Color					
L*	48 h	35.74	30.50	1.93	0.004
a*	48 h	10.55	6.94	0.77	0.000
b*	48 h	10.90	6.42	1.29	0.005
C*	48 h	15.25	9.74	1.21	0.000
*h°*	48 h	46.10	39.50	3.50	0.127

DFD: dark, firm, and dry; C*: chroma; *h°*: hue angle. *p*-value < 0.05 indicates significant differences between CONTROL and DFD samples.

**Table 2:** Mapping statistics.

	Samples	Total Reads	Total Small RNA Reads (%)	Small RNAs (%)								
				Protein Coding RNAs	rRNAs	snRNAs	snoRNAs	miRNAs	lncRNAs	misc-RNAs	scaRNAs	sRNAs
CONTROL (5.4 ≤ pH_24_ ≤ 5.6)	1B	14,805,539	44	33.14	3.72	1.89	14.04	20.17	10.78	16.01	0.137	0.007
	2B	7,137,747	52	41.18	3.61	2.01	12.10	18.02	8.84	14.02	0.099	0.013
	3B	11,939,843	45	29.02	4.96	1.53	14.79	29.92	4.69	14.88	0.108	0.000
	4B	10,511,703	48	52.67	2.00	2.37	8.41	11.91	13.11	9.38	0.071	0.004
	5B	10,419,003	48	37.32	4.73	1.06	12.72	26.53	4.83	12.64	0.093	0.003
DFD (pH_24_ > 6.2)	1A	12,854,015	52	66.83	1.47	0.57	1.54	2.14	21.73	5.65	0.018	0.001
	2A	11,807,132	44	34.61	3.72	0.87	6.93	17.08	20.81	15.81	0.056	0.004
	3A	12,273,319	47	57.24	2.03	0.70	3.13	5.31	21.01	10.50	0.037	0.002
	4A	16,874,438	51	81.35	1.12	0.63	1.18	0.87	11.21	3.57	0.022	0.001
	5A	11,361,830	50	59.33	2.59	2.40	3.80	7.39	16.14	8.21	0.049	0.001

rRNA: ribosomal ribonucleic acid; snRNA: small nuclear RNA; snoRNA: small nucleolar RNA; miRNA: microRNA; lncRNA: large non-coding RNA; misc-RNA: miscellaneous RNA; scaRNA: small Cajal body-specific RNA; sRNA: bacterial small RNA. Total reads refer to the number of deduplicated reads that were used as input for the mapping step to miRBase and all other small RNA databases. All columns with the unit (%) depict the fraction of the column total reads.

**Table 3:** Differences in the abundance of small RNA classes depending on the pH_24_ sample type (CONTROL vs. DFD) (mean ± standard error (SEM)).

Small RNA Class	CONTROL	DFD	SEM	*p*-Value
rRNA	3.81	2.19	0.695	0.049
Protein coding RNA	38.67	59.87	8.610	0.048
snRNA	1.78	1.04	0.411	0.118
snoRNA	12.42	3.32	1.510	0.000
miRNA	21.31	6.56	4.281	0.009
lncRNA	8.45	18.19	2.597	0.006
misc-RNA	13.39	8.75	2.405	0.101
scaRNA	0.10	0.04	0.013	0.001
sRNA	0.006	0.002	0.002	0.168

rRNA: ribosomal ribonucleic acid; snRNA: small nuclear RNA; snoRNA: small nucleolar RNA; miRNA: microRNA; lncRNA: large non-coding RNA; misc-RNA: miscellaneous RNA; scaRNA: small Cajal body-specific RNA; sRNA: bacterial small RNA.

**Table 4:** Differentially expressed miRNAs between CONTROL and DFD.

miRNA	Log_2_ Fold Change	*p*-Value	FDR *p*-Value	Bonferroni
*bta-miR-27a-5p*	3.447	4.5 × 10^−19^	1.9 × 10^−16^	3.1 × 10^−16^
*bta-miR-2332* *	3.735	1.6 × 10^−9^	3.5 × 10^−7^	1.1 × 10^−6^
*bta-miR-12034*	5.117	1.9 × 10^−8^	2.8 × 10^−6^	1.3 × 10^−5^
*bta-miR-2411-3p* *	3.718	3.92 × 10^−8^	4.3 × 10^−6^	2.7 × 10^−5^
*bta-miR-11980*	4.035	1.3 × 10^−7^	1.1 × 10^−5^	8.6 × 10^−5^
*bta-miR-11987*	3.535	5 × 10^−7^	3.6 × 10^−5^	3.4 × 10^−4^
bta-miR-1246 *	2.236	1.3 × 10^−6^	8.3 × 10^−5^	9.1 × 10^−4^
*bta-miR-23b-5p* *	1.979	2 × 10^−6^	1.1 × 10^−4^	1.4 × 10^−3^
*bta-miR-12030*	3.339	5.2 × 10^−6^	2.5 × 10^−4^	3.6 × 10^−3^
*bta-miR-193a-5p*	1.000	7.2 × 10^−6^	2.9 × 10^−4^	4.9 × 10^−3^
*bta-miR-11972*	4.973	7.5 × 10^−6^	2.9 × 10^−4^	5.2 × 10^−3^
*bta-miR-2887*	2.793	2.7 × 10^−5^	9.6 × 10^−4^	1.8 × 10^−2^

* These miRNAs were validated using RT-qPCR.

**Table 5:** RT-qPCR-validated miRNAs and some of their potential gene targets in functions related to oxidative damage and cell death (apoptosis).

microRNA	Symbol	Gene Name
*miR-2332*	HSPA12B	70 kDa heat shock protein 12B
	HSPBAP1	HSPB (27 kDa heat shock) associated protein 1
	HSPA4	70 kDa heat shock protein 4
	HSBP1	Heat shock factor binding protein 1
	HSPH1	105/110 kDa heat shock protein 1
	DNAJA1	DnaJ (Hsp40) homolog, subfamily A, member 1
	DNAJB9	DnaJ (Hsp40) homolog, subfamily B, member 9
	DNAJC10	DnaJ (Hsp40) homolog, subfamily C, member 10
*miR-2411-3p*	GSR	Glutathione reductase
	GPX5	Glutathione peroxidase 5
	SERP1	Stress-associated endoplasmic reticulum protein 1
	CLU	Clusterin
	HSPA2	70 kDa heat shock protein 2
	TP53AIP1	Tumor protein p53-regulated apoptosis-inducing protein 1
	CASP2	Caspase 2
	CASP9	Caspase 9

## Data Availability

The original contributions presented in the study are included in the article/Supplementary Material, further inquiries can be directed to the corresponding author.

## Supplementary Materials

The following supporting information can be downloaded at: https://www.mdpi.com/article/10.3390/foods13060960/s1, Table S1: Ranking of the most stable miRNAs according to the Normfinder algorithm.
