# Supplementary material for: Exploring the miRNAs Profile in Dark-Cutting Beef
Source: Foods. 2024 Mar 21;13(6):960. doi: 10.3390/foods13060960 (PMC10970431; doi:10.3390/foods13060960)
Supplement: Supplementary file 1 [file foods-13-00960-s001.zip › foods-2902281-supplementary.pdf]

Table S1. Ranking of the most stable miRNAs according to the Normfinder algorithm.

**miRNA**	**C_t (CONTROL)** (mean ± SEM)	**C_t (DFD)** (mean ± SEM)	**p-value**
**mmu-let-7d**	28.55 ± 0.7	28.96 ± 0.8	0.313
**hsa-miR-125b**	24.79 ± 0.7	25.03 ± 0.9	0.580
**bta-miR-425-5p**	29.93 ± 0.8	30.25 ± 0.9	0.486
**bta-miR-660**	27.87 ± 0.8	28.22 ± 0.8	0.430
**hsa-miR-148b**	28.93 ± 0.6	29.02 ± 0.8	0.814
**hsa-miR-151-3p**	28.55 ± 0.7	28.96 ± 0.8	0.640
**hsa-miR-10b**	24.67 ± 0.7	25.04 ± 0.9	0.417
**bta-miR-342**	26.41 ± 0.6	26.76 ± 0.8	0.348

DFD: dark, firm and dry.
